# Difficulty Standing on the Tiptoes? Think of an Epiconus Syndrome: A Case Report and a Review of the Pathobiology of the Conus and Epiconus

**DOI:** 10.7759/cureus.12724

**Published:** 2021-01-15

**Authors:** Hassan Kesserwani

**Affiliations:** 1 Neurology, Flowers Medical Group, Dothan, USA

**Keywords:** bilateral limb weakness, spinal cord injury

## Abstract

The motor deficits, urogenital dysfunction and perineal numbness of the conus medullaris syndrome are well known. Less well known is the disease of the epiconus, the spinal cord immediately above the conus medullaris. The disease is quite unique with ankle plantar-flexion weakness that usually exceeds ankle dorsi-flexion weakness. The epiconus syndrome can present with both upper and lower motor neuron findings and manifest unique findings on nerve conduction/electromyography studies. Intriguingly, lumbo-sacral corticospinal tract disease can involve trans-synaptic degeneration of the anterior horn motor neurons and lead to acute denervation, as recorded with electromyography. The conus medullaris also contains Onuf’s nucleus, which controls penile erection, ejaculation, the external urethral and the external anal sphincter and is the basis of the bulbo-cavernosus reflex. Extension of a lesion from the epiconus to the conus can lead to urogenital dysfunction. We seize upon a case of an epiconus syndrome in order to outline some of these fascinating observations including the pathobiology of the conus and epiconus. In order to understand the epiconus, one must be versed with the conus medullaris.

## Introduction

The tip of the conus medullaris ends variably between thoracic vertebral body T11 and lumbar vertebral body L3, most commonly at L1. This variability is due to the differential lengthening of the spinal cord and vertebral column during embryonic development [1]. The conus medullaris contains neural tissue from sacral cord segments S2, S3, S4, S5 and coccygeal 1. Superiorly, the epiconus extends from lumbar cord segments L3, L4, L5 and sacral cord segment S1. This usually corresponds to vertebral levels T12 and L1. Of note, the filum terminale contains filaments from the epiconus and conus, lumbar cord segments L3 to coccygeal cord segment 1.

Damage of the conus medullaris can lead to urogenital, sensory and/or motor dysfunction. Urogenital dysfunction includes bladder atonia with stress incontinence if the lesion is in the conus. If the lesion is in the epiconus, one can get bladder hypertonus and spincter dyssynergia (bladder retention). Sexual dysfunction, such as erectile dysfunction (achieving an erection), is common. Classically, anesthesia of the perineum is saddle shaped due to concentric dermatomal distribution (sacral cord segment S3, S4 and S5) and may involve anal sphincter atonia and bowel leakage. Due to sacral segment S2 involvement, the weakness of the lower legs involves plantar flexion, more than dorsi-flexion, with difficulty tiptoeing. With compressive lesions of the epiconus, the peripheral disposition of the sacral fibers compared to the lumbar fibers in the corticospinal tracts may explain preferential weakness of the plantar flexors [2].

Onuf's nucleus is located in the anterior horn of the spinal cord, Rexed lamina IX. These anterior horn cells are the origin of the pudendal nerve and they originate variably from neural cord segments S1, S2 and S3. They supply motor neurons and control the striated muscles of the anal and urethral sphincter, controlling defecation and micturition, respectively. Onuf's nucleus also controls the bulbo-cavernosus muscle, which is involved in penile erection and ejaculation. Interestingly, Onuf's nucleus cell number is reduced in Parkinson's disease and Shy-Drager syndrome, explaining bladder dysfunction in these diseases. It is spared in amyotrophic lateral sclerosis and spinal muscular atrophy. This is an intriguing observation as the neurons of Onuf's nucelus have peptidergic innervation, rendering them an autonomic phenotype, and autonomic dysfunction is common with the latter two diseases [3]. This reflex can be measured by the bulbo-cavernosus reflex (BCR).

The BCR is an S2, S3 and S4 spinal cord reflex mediated by the pudendal nerve, whose anterior horn motor cells reside in Onuf’s nucleus. Both the afferent and efferent reflex arcs reside in the pudendal nerve. The reflex is elicited by pinching the glans of the penis or clitoris. It is measured by contraction of the bulbo-cavernosus at the base of the penis, contraction of the vaginal sphincter and external anal sphincter. Therefore, the BCR measures the integrity of the conus medullaris [4].

In the Case Presentation, we outline a case of an epiconus spondylotic compression with medullary hyperintensity on imaging studies, with greater ankle plantar-flexion than dorsi-flexion weakness. In the Discussion section, we explain this pattern of weakness, outline the differences between conus medullaris and epiconus spinal cord lesions, outline the blood supply of the spinal cord including the conus medullaris (the basket conus) and discuss the pathophysiology and pathobiology of epiconus compressive lesions. Tumors and ischemic infarcts of the conus and epiconus are outside the scope of this article.

## Case presentation

We describe the case of a 32-year-old young woman who presents with bilateral symmetric distal leg weakness, who noted difficulty standing on her tiptoes when reaching upwards high on her kitchen cabinet. She denied any back pain, bladder or rectal dysfunction. The weakness was slowly progressive over the course of two years. Of note, she denied any arm or bulbar weakness (dysphonia, dysarthria, choking, dysphagia or dyspnea).

Her past medical history is unremarkable.

On examination, we will list the pertinent findings. Her gait was characterized by dragging her legs, with inability to stand on the tip-toes. Heel walking was achieved with relative ease. Romberg sign was absent.

We record bilateral lower extremity power on a Medical Research Council Scale (MRC); graded from 0 to 5 (Table 1).

**Table 1:** Power of bilateral lower extremity muscles. Note ankle plantar flexion more than ankle dorsi-flexion weakness.

MUSCLE	RIGHT LEG	LEFT LEG
Ilio-psoas	5	5
Hip adductors	5	5
Hip abductors	5	5
Quadriceps	5	5
Hamstrings	5	5
Ankle dorsi-flexion	4	4
Ankle plantar flexion	2	2

The deep tendon reflexes in the upper and lower extremities were all preserved with the exception of bilateral absent ankle jerks. Mild bilateral calf-muscle atrophy was noted. Sensation in the toes was preserved to joint position sense, vibration, touch, pressure and pinprick. The rest of the neurological examination including cerebellar function and cranial nerves was entirely normal.

**Figure 1:**
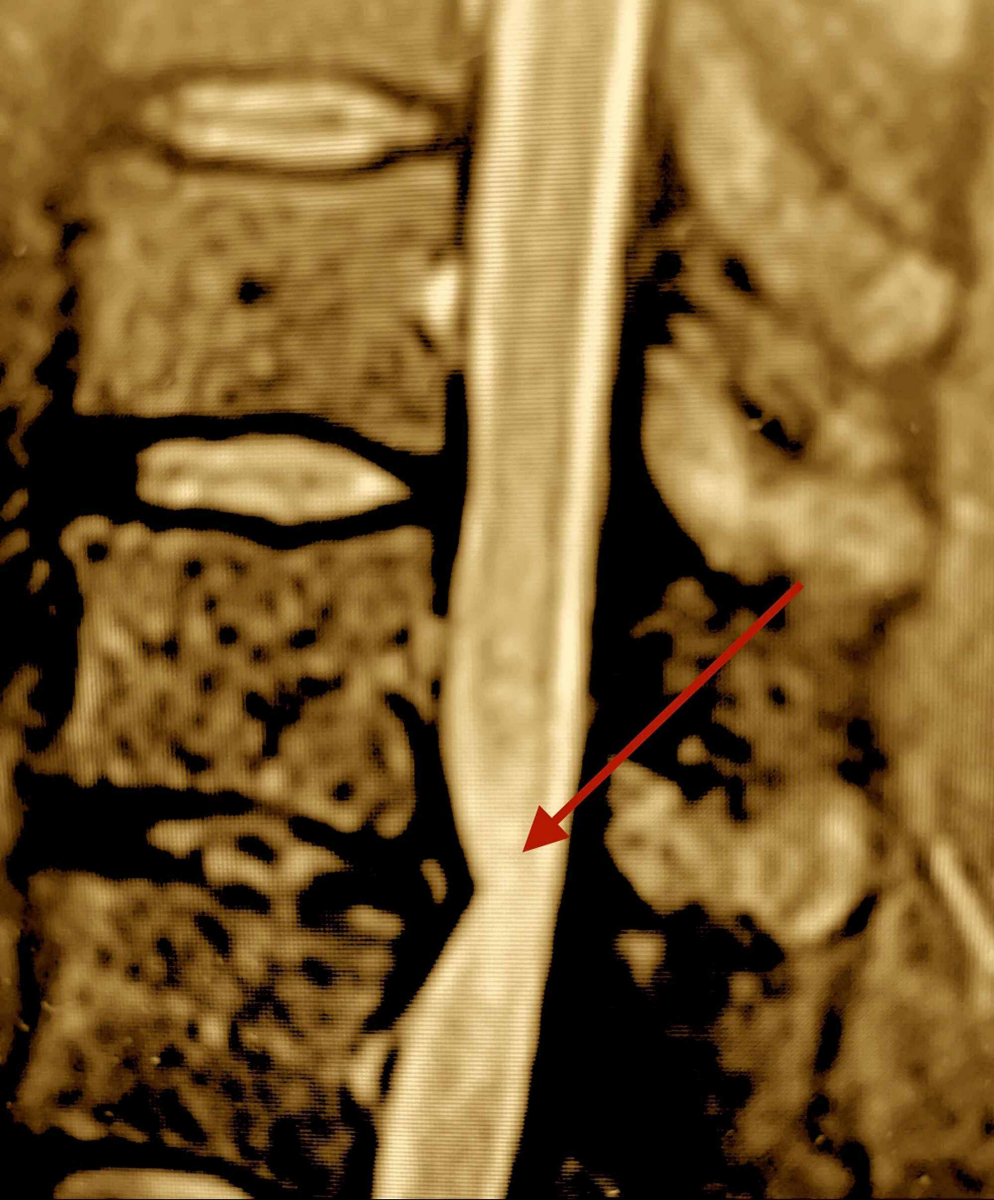
STIR-MRI thoracic cord: epiconus medullaris compression with edema at T12-L1. STIR signal abnormality in the medulla of the spinal cord indicates inflammation or ischemia (red arrow). STIR-MRI: Short-T1 inversion recovery-magnetic resonance imaging Thoracic (T) and lumbar (L).

A sagittal magnetic resonance imaging (MRI) with short T1-inversion recovery (STIR) study revealed epiconus medullaris compression at the thoraco-lumbar T12-L1 level vertebral level, with signal hyperintensity in the medulla of the epiconus medullaris (Figure 1).

A nerve conduction study (NCS) revealed bilateral absent peroneal motor amplitudes with bilateral preserved tibial motor compound muscle action potential (CMAP amplitudes); an unexpected finding given greater ankle plantar-flexion than ankle dorsi-flexion weakness. The motor velocities were normal. The bilateral peroneal and sural sensory amplitudes were preserved. The tibial H-reflexes were absent bilaterally, as expected.

**Figure 2:**
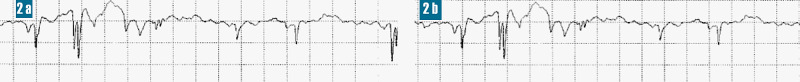
Electromyography - acute florid denervation with runs of positive sharp waves involving (A) right gastrocnemius and (B) right short head of the biceps femoris.

An electromyogram (EMG) revealed acute florid denervation of the gastrocnemius and the short head of the biceps femoris bilaterally (Figure 2).

The peroneal nerve-innervated peroneus longus and tibialis anterior revealed moderately severe chronic denervation.

This odd electrophysiological pattern needs further elaboration. The absent bilateral tibial H-reflexes and preserved sensory nerve action potential concur with sacral S1 nerve root disease as does the acute florid denervation of the gastrocnemius and short head of the biceps femoris bilaterally. However, the absent peroneal nerve CMAPs, but preserved tibial motor CMAPs, can be explained by cortico-spinal tract involvement with trans-synaptic denervation. This may explain the weakness of plantar flexion with acute denervation and preserved CMAPs. In the next section, we present evidence to support this hypothesis.

Meanwhile, the patient was referred to neurosurgery for thoraco-lumbar cord decompression.

## Discussion

The spinal cord is supplied by longitudinal blood vessels, a solitary anterior spinal artery (ASA) and a dual posterior spinal artery (PSA). The ASA and PSA arise from the vertebral arteries, the PSA occasionally from the posterior inferior cerebellar artery (PICA). The ASA sends perforators into the central spinal cord. The ASA and PSA both send circumferential pial arteries and perforators into the spinal cord proper. The ASA and PSA are fed and reinforced by segmental arteries, also referred to as feeders. The feeders divide into ascending and descending branches. There is one outstanding feeder; the artery of Adamkiewicz, formerly known as the great spinal artery (GSA). In the vast majority of cases, the GSA enters the left ventral roots between thoracic T8 and T11 and feeds the ASA, supplying the distal cord; this is the caudal variant. Sometimes the GSA arrives as multiple feeders, entering between thoracic T10 and lumbar L1. When the feeder supplies the proximal cord, the GSA enters the T10 dorsal root and supplies the PSA [5].

**Figure 3:**
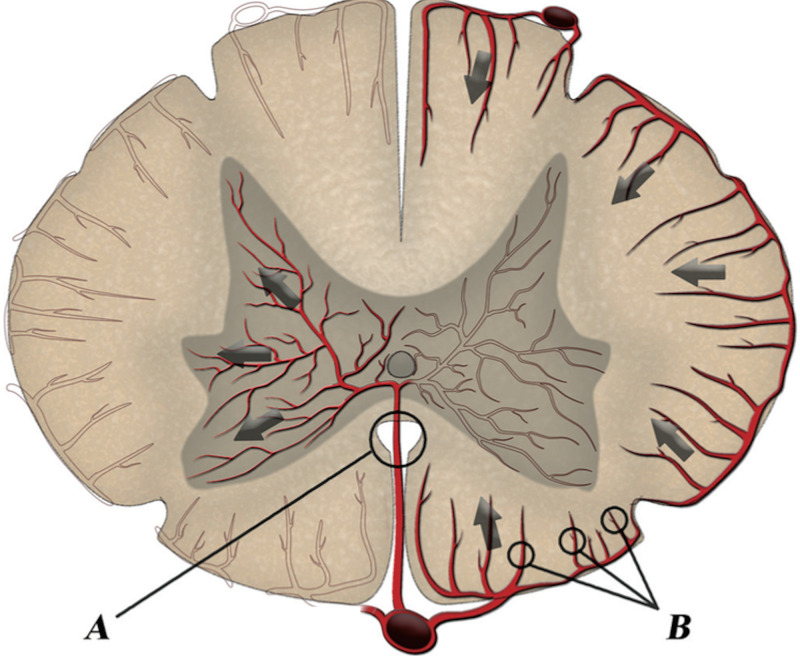
Blood supply of the spinal cord. A represents the perforators supplying blood flow to the anterior two-third of the spinal cord in a centrifugal manner and B represents perforators from the circumferential arteries penetrating the spinal cord in a centripetal manner.

Returning to the ASA, the perforators enter the ventral roots, which they supply, and feed the anterior two-thirds of the spinal cord. They divide into longitudinal branches, which themselves issue circumferential branches. The latter penetrate the lateral funiculi of the spinal cord. The direction of blood flow in the ASA is from proximal to distal. It is interesting that the ASA widens when joined by the GSA, hemodynamically explaining the distal direction of flow by Bernoulli's theorem; flow from narrow caliber high-pressure system to a wider caliber low-pressure system. On the posterior surface of the spinal cord, the dynamics of blood flow are entirely different. At the cervical and thoracic level, blood flow is from proximal to distal. However, at the lumbar and sacral levels, blood flow is from distal to proximal. At the fifth sacral level, S5, in the posterior nerve roots, two terminal branches of the ASA, anastamose with each PSA. This blood flow is reinforced by segmental branches at the lumbar level. This pattern rendered the blood flow on the posterior surface of the spinal cord as end arteries at the distal thoracic segment. This pattern of flow is similar at the anterior spinal cord, before the entry of the GSA, and represents water-shed zones. Therefore, any compressive lesion at a site distant to this water-shed zone may lead to ischemic injury far from the site of compression. In this manner, an upper cervical cord lesion may cause pain and wasting of the muscles of the hands (Figure 3) [6].

The circumferential branches are very well developed around the conus medullaris. The blood supply of the conus medullaris arises from one or two feeders from the ASA anastamosing with the PSA, the so-called arterial basket of the conus medullaris. This system is bilateral and sends perforators into the conus medullaris [7].

The branches from the ASA and PSA are supplying the spinal cord number between 15 and 34. They go by various names including medullary arteries, radicular arteries and radiculo-medullary arteries. 

**Figure 4:**
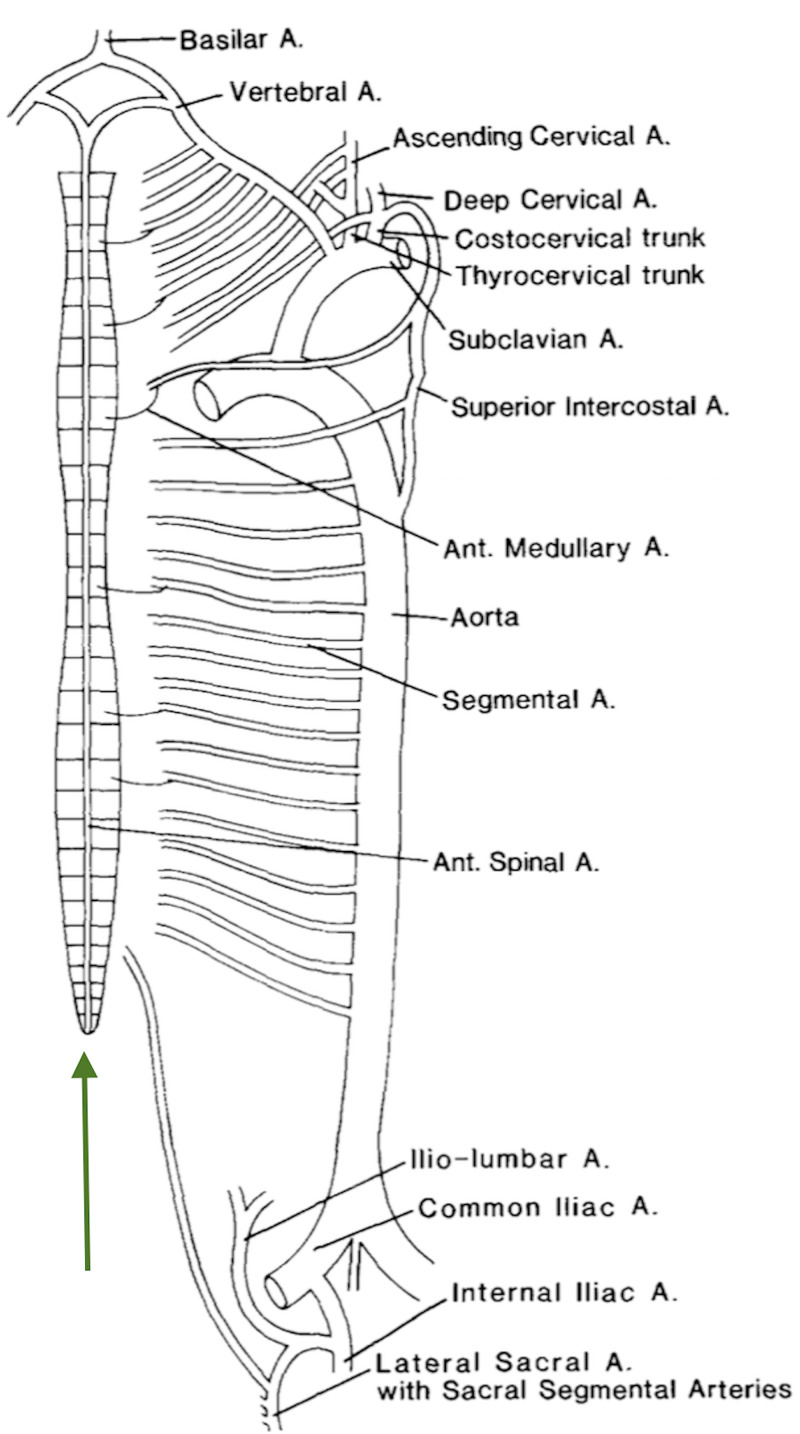
Segmental arteries to the spinal cord and anterior and posterior cerebral arteries. Spinal cord (green arrow). A - Artery Ant - anterior

There are 31 pairs of segmental arteries. Only a few supply the spinal cord directly. Instead, they supply the ASA and PSA, arising from the vertebral arteries, ascending cervical arteries (thyrocervical trunk), deep cervical arteries and superior intercostal artery (both from the costocervical trunk), aorta, iliolumbar artery and lateral sacral artery (Figure 4) [8].

Rarely, the conus medullaris is supplied by a branch from the internal iliac artery or the iliolumbar artery, the aptly named "cone artery". This variant also known as the Desproges-Gotteron artery ascends along the L5 and S1 nerve roots and may lead to the conus syndrome with compression from spinal disc disease [9]. Overall, the rich blood supply of the spinal cord with overlapping territories and dense anastomotic network renders it resistant to ischemic injury.

The pathophysiology of spondylotic myelopathy is distinct from traumatic spinal cord injury. With the former, there is evidence of radiculo-medullary artery ischemia due to stenosis of the inter-vertebral foramen. The circumferential branches from the ASA and the perforators from the pial plexus are stretched in cadavers with spondylotic myelopathy. There is also histopathologic evidence of ischemic injury to both the gray and white matter of the spinal cord [10]. Other than direct compression of the spinal cord, venous occlusion may also lead to T2- and STIR MRI signal hyperintensity of the spinal cord with a "snake-eyes" appearance on axial cuts. This may explain the medullary hyperintensities on imaging studies and the lack of complete recovery following decompressive surgery [11].

**Figure 5:**
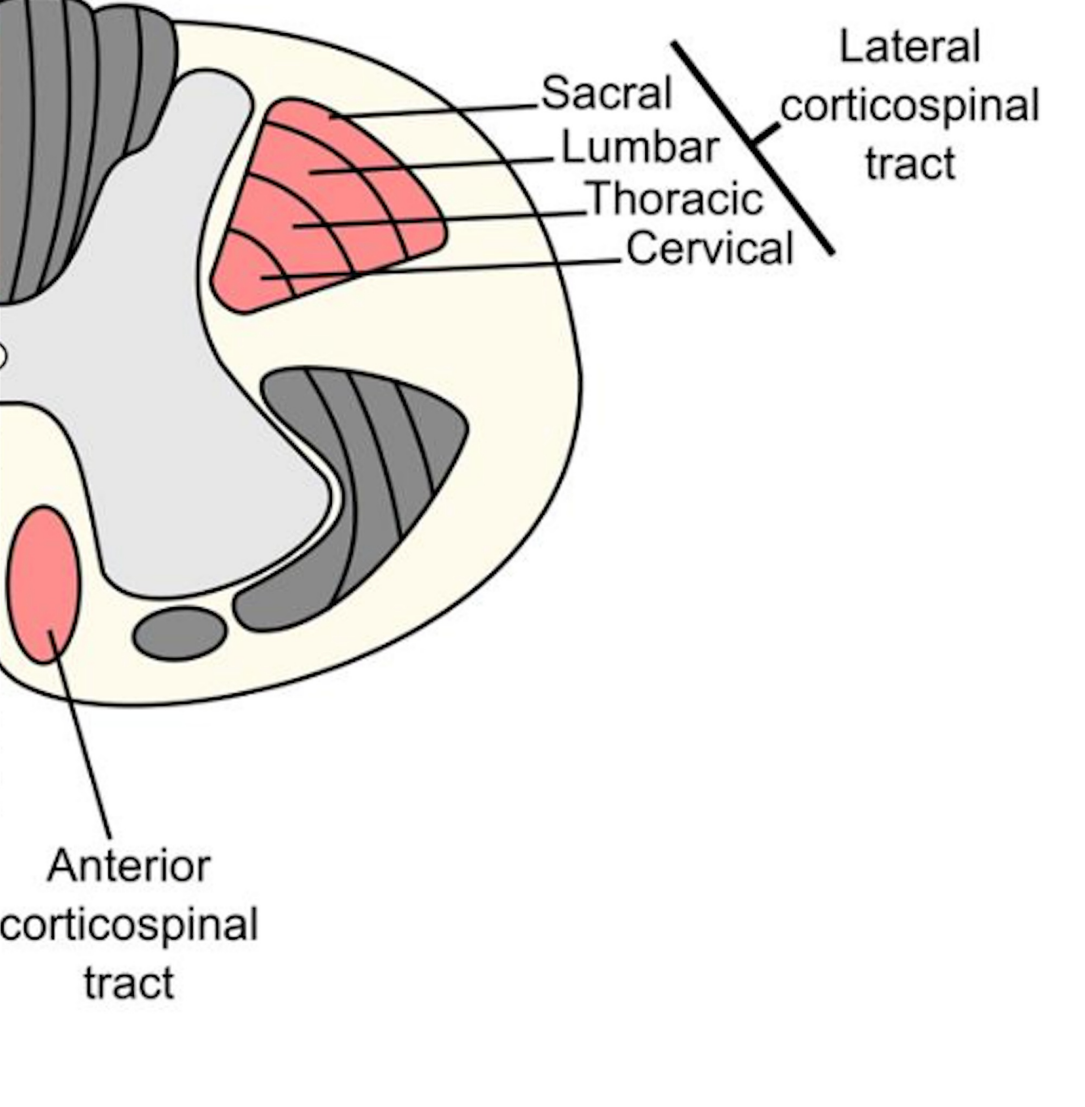
Topography of the lateral corticospinal tract demonstrating the peripheral location of the sacral fibers and predisposition to compression.

Weakness of plantar flexion of the ankles usually exceeds weakness of dorsi-flexion, as the spinal cord compression or disease involves the S2 sacral segments, the nerve root to the gastrocnemius and soleus. With an epiconus lesion, the topography of the sacral fibers of the cortico-spinal tract is more laterally displaced, with respect to the lumbar fibers (Figure 5) [12].

The conus medullaris syndrome is a lower motor neuron syndrome and the epiconus syndrome is usually an upper motor neuron syndrome, but due to trans-synaptic degeneration, the epiconus syndrome may present with lower motor neuron signs (Table 2) [13,14].

**Table 2:** Table distinguishing clinical features of the conus medullaris syndrome and the epiconus syndrome. BCR - Bulbo-cavernosus reflex EMG - Electromyogram CMAP - Compound muscle action potential

CONUS MEDULLARIS SYNDROME	EPICONUS SYNDROME
Faccid weakness	Spastic weakness
Areflexia	Usually hyper-reflexia, but can be areflexia
Absent BCR, especially with more severe disease	Normal BCR
Denervation on EMG	Rarely denervation, only with trans-synaptic denervation
Diminished peroneal and tibial CMAP	Usually normal peroneal and tibial CMAP
Bladder atonia	Detrusor hyper-reflexia with sphincter dysynergia

A retrospective review of 15 patients with the epiconus syndrome, Toribatake et al. identified lower motor neuron, upper motor neuron and mixed upper and lower motor neuron findings. Ten out of the 15 patients had a co-existent conus medullaris syndrome with sphincter dysfunction. Most patients had a compressive spondylotic disease. The findings are summarized below (Table 3) [15]. 

**Table 3:** Clinical findings of 15 patients with the epiconus syndrome. L - Lumbar S - Sacral

CLINICAL FINDING	NUMBER OF PATIENTS
Lower motor neuron signs (hyporeflexia, areflexia)	13
Upper motor neuron sign (hyper-reflexia)	3
Mixed upper and lower motor neuron signs	Not disclosed, possibly two patients
Sensory symptoms (mostly L5 and S1)	14
Conus medullaris syndrome (bladder dysfunction and possibly perineal numbness)	5
Bilateral leg weakness	6
Radicular symptoms	4
Gastocnemius atrophy	9
Ankle dorsi-flexion weakness	7

Unfortunately, this study does not compare the differential pattern of weakness between the ankle-plantar and ankle dorsi-flexors.

## Conclusions

The epiconus syndrome is a fascinating neuro-anatomic disorder characterized by unique features; ankle plantar-flexion more than ankle dorsi-flexion weakness and lower, upper and possibly mixed upper and lower motor neuron findings. It also has seemingly odd NCS/EMG findings, which may be explained by trans-synaptic degeneration. Extension into the conus medullaris may lead to sensory symptoms and urogenital dysfunction. Integral to the understanding of the epiconus is the conus medullaris, Onuf's nucleus, and somatotopic division of the corticospinal tracts and their blood supply, all of which we address in detail in this case report. Future studies should be aimed at characterizing the pattern of the weakness of the lower extremities.
